# 1-(2-Methyl-5-nitro-1*H*-imidazol-1-yl)acetone

**DOI:** 10.1107/S1600536813006569

**Published:** 2013-03-16

**Authors:** Sammer Yousuf, Khalid M. Khan, Frazana Naz, Shahanaz Perveen, Ghulam A. Miana

**Affiliations:** a H.E.J. Research Institute of Chemistry, International Center for Chemical and Biological Sciences, University of Karachi, Karachi 75270, Pakistan; b PCSIR Laboratories Complex Karachi, Shahrah-e-Dr Salimuzzaman Siddiqui, Karachi 75280, Pakistan; c Ripha Insititue of Pharmaceutical Sciences, Ripha International University, 7th Avenue G-7/4 Islamambad, Pakistan

## Abstract

In the mol­ecule of the title compound, C_7_H_9_N_3_O_3_, the nitro and carbonyl groups are tilted with respect to the imidazole ring by 9.16 (6) and 65.47 (7)°, respectively. Neighbouring chains are linked *via* C—H⋯N and C—H⋯O hydrogen bonds forming two-dimensional slab-like networks lying parallel to (01-1).

## Related literature

For the anti­biotic properties of metronidazole and mecnidazole, see: Lin *et al.* (2012 ▶); Almirall *et al.* (2011 ▶); Zhang *et al.* (2011 ▶). For the crystal structure of related imidazoles, see: Yousuf *et al.* (2012 ▶); Zeb *et al.* (2012 ▶).
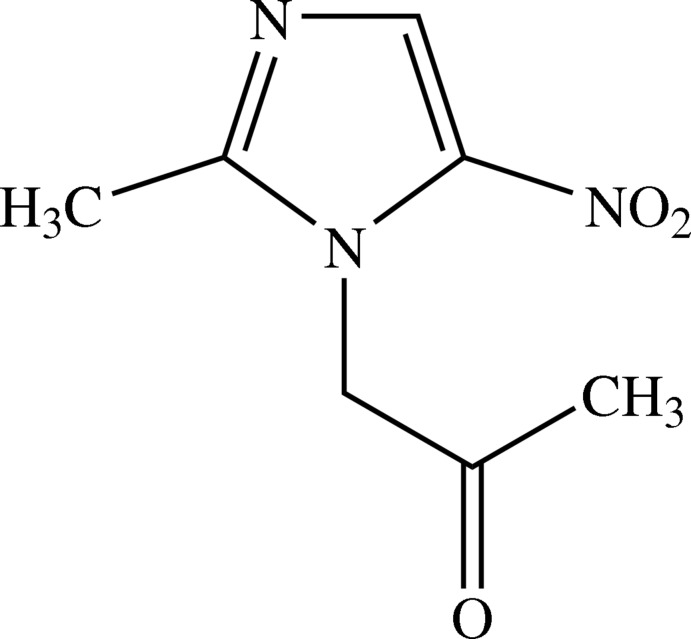


## Experimental

### 

#### Crystal data

C_7_H_9_N_3_O_3_
*M* *_r_* = 183.17
Monoclinic, 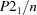

*a* = 4.7548 (4) Å
*b* = 12.3971 (9) Å
*c* = 14.8580 (11) Å
β = 97.350 (2)°
*V* = 868.62 (12) Å^3^
*Z* = 4
Mo *K*α radiation
μ = 0.11 mm^−1^
*T* = 273 K
0.52 × 0.33 × 0.24 mm

#### Data collection

Bruker SMART APEX CCD area-detector diffractometer
Absorption correction: multi-scan (*SADABS*; Bruker, 2000 ▶) *T*_min_ = 0.944, *T*_max_ = 0.974
5030 measured reflections
1614 independent reflections
1328 reflections with *I* > 2σ(*I*)
*R*_int_ = 0.019

#### Refinement

*R*[*F* ^2^ > 2σ(*F* ^2^)] = 0.041
*wR*(*F* ^2^) = 0.122
*S* = 1.06
1614 reflections
120 parameters
H-atom parameters constrained
Δρ_max_ = 0.19 e Å^−3^
Δρ_min_ = −0.15 e Å^−3^

### 

Data collection: *SMART* (Bruker, 2000 ▶); cell refinement: *SAINT* (Bruker, 2000 ▶); data reduction: *SAINT*; program(s) used to solve structure: *SHELXS97* (Sheldrick, 2008 ▶); program(s) used to refine structure: *SHELXL97* (Sheldrick, 2008 ▶); molecular graphics: *SHELXTL* (Sheldrick, 2008 ▶); software used to prepare material for publication: *SHELXTL*, *PARST* (Nardelli, 1995 ▶) and *PLATON* (Spek, 2009 ▶).

## Supplementary Material

Crystal structure: contains datablock(s) global, I. DOI: 10.1107/S1600536813006569/rz5048sup1.cif

Structure factors: contains datablock(s) I. DOI: 10.1107/S1600536813006569/rz5048Isup2.hkl

Supplementary material file. DOI: 10.1107/S1600536813006569/rz5048Isup3.cml

Additional supplementary materials:  crystallographic information; 3D view; checkCIF report

## Figures and Tables

**Table 1:** Hydrogen-bond geometry (Å, °)

*D*—H⋯*A*	*D*—H	H⋯*A*	*D*⋯*A*	*D*—H⋯*A*
C2—H2*B*⋯N2^i^	0.93	2.56	3.361 (2)	144
C5—H5*B*⋯O2^ii^	0.97	2.57	3.527 (2)	167
C7—H7*B*⋯O3^iii^	0.96	2.49	3.340 (2)	147

Symmetry codes: (i) 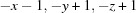; (ii) 
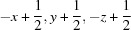
; (iii) 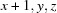.

**Fig. 1.**
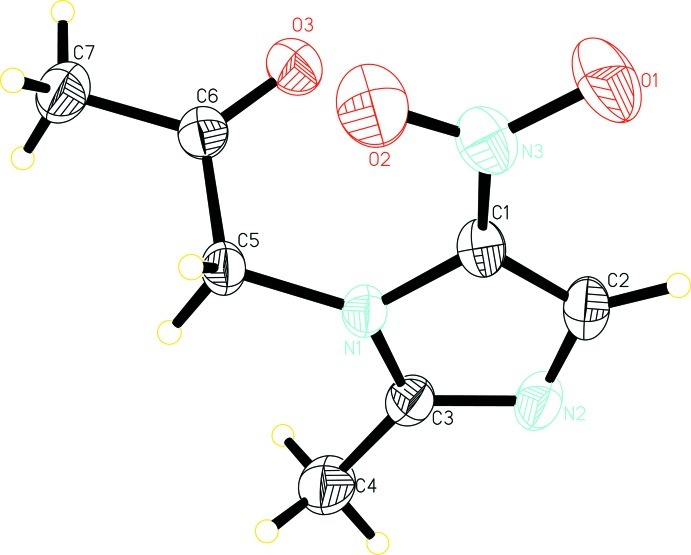
The molecular structure of the title compound with displacement ellipsoids drawn at 30% probability level.

**Fig. 2.**
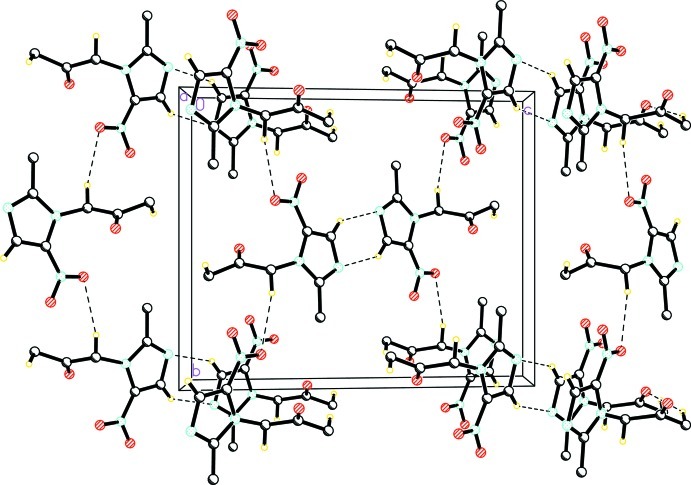
The crystal packing of the title compound. Intermolecular hydrogen bonds are shown as dashed lines.

## supplementary crystallographic information

### Comment

Imidazole nuclei containing metronidazole and mecnidazole are widely used antibiotics, known to be effective against anaerobic microorganisms. These drugs employed to cure amoebiasis (Almirall *et al.*, 2011) and protozoal infections (Lin *et al.*, 2012). Secnidazoles is also reported to have anti-inflammatory and urease inhibiton activites (Zhang *et al.*, 2011). The title compound is a derivative of secnidazole obtained during our attempts to make more effective structure analogues of this important antibacterial drug.

The structure of the title compound (Fig. 1) is similar to that of our previously published compound 2-(2-methyl-5-nitro-1*H*-imidazol-1-yl)-ethyl methanesulfonate (Zeb *et al.*, 2012) with the difference that the ethyl methanesulfonate attached to the imidazole ring is replaced by an acetone (O3/C5—C7) group. Bond length and angles were found to be similar to those reported for related structures (Yousuf *et al.*, 2012). In the crystal, molecules are linked by C2—H2B···N2, C5—H5B···O2 and C7—H7B···O3 intermolecular interactions (Table 1) to form a three-dimensional network (Fig. 2).

### Experimental

Periodic acid (2.8 mmol, 0.64 g), pyridinium chlorochromate (PCC, 4 mol%) were suspended in acetonitrile (20 ml) and stirred vigorously for five minutes. The mixture was allowed to cool on an ice-salt bath followed by the addition of secnidazole (2.7 mmol, 0.50 g) and allowed to stir for 36 h at ambient temperature. After the completion of the reaction [TLC analysis], the reaction mixture was washed with brine/water (1:1 *v*/*v*), saturated aqueous Na_2_SO_3_ solution, dried (Na_2_SO_4_) and filtered. The filtrate was evaporated *in vacuum* to afford off-white crystals which were washed and recrystalized by dissolving in petroleum ether to obtained colorless crystals of the title compound (0.32 g, 64% yield) found suitable for single-crystal X-ray diffraction analysis.

### Refinement

H atoms of methyl, methylene and methine carbon atoms were positioned geometrically with C—H = 0.93–0.96 Å and constrained to ride on their parent atoms with *U*_iso_(H)= 1.2*U*_eq_(C) or 1.5*U*_eq_(C) for methyl H atoms. A rotating group model was applied to the methyl group.

### Crystal data

**Table:**

C_7_H_9_N_3_O_3_	*F*(000) = 384
*M**_r_* = 183.17	*D*_x_ = 1.401 Mg m^−^^3^
Monoclinic, *P*2_1_/*n*	Mo *K*α radiation, λ = 0.71073 Å
Hall symbol: -P 2yn	Cell parameters from 1790 reflections
*a* = 4.7548 (4) Å	θ = 2.8–26.7°
*b* = 12.3971 (9) Å	µ = 0.11 mm^−^^1^
*c* = 14.8580 (11) Å	*T* = 273 K
β = 97.350 (2)°	Block, colorless
*V* = 868.62 (12) Å^3^	0.52 × 0.33 × 0.24 mm
*Z* = 4	

### Data collection

**Table:**

Bruker SMART APEX CCD area-detector diffractometer	1614 independent reflections
Radiation source: fine-focus sealed tube	1328 reflections with *I* > 2σ(*I*)
Graphite monochromator	*R*_int_ = 0.019
ω scan	θ_max_ = 25.5°, θ_min_ = 2.2°
Absorption correction: multi-scan (*SADABS*; Bruker, 2000)	*h* = −5→5
*T*_min_ = 0.944, *T*_max_ = 0.974	*k* = −14→15
5030 measured reflections	*l* = −14→17

### Refinement

**Table:**

Refinement on *F*^2^	Primary atom site location: structure-invariant direct methods
Least-squares matrix: full	Secondary atom site location: difference Fourier map
*R*[*F*^2^ > 2σ(*F*^2^)] = 0.041	Hydrogen site location: inferred from neighbouring sites
*wR*(*F*^2^) = 0.122	H-atom parameters constrained
*S* = 1.06	*w* = 1/[σ^2^(*F*_o_^2^) + (0.0591*P*)^2^ + 0.2124*P*] where *P* = (*F*_o_^2^ + 2*F*_c_^2^)/3
1614 reflections	(Δ/σ)_max_ < 0.001
120 parameters	Δρ_max_ = 0.19 e Å^−^^3^
0 restraints	Δρ_min_ = −0.15 e Å^−^^3^

### Special details

**Table:**

Geometry. All e.s.d.'s (except the e.s.d. in the dihedral angle between two l.s. planes) are estimated using the full covariance matrix. The cell e.s.d.'s are taken into account individually in the estimation of e.s.d.'s in distances, angles and torsion angles; correlations between e.s.d.'s in cell parameters are only used when they are defined by crystal symmetry. An approximate (isotropic) treatment of cell e.s.d.'s is used for estimating e.s.d.'s involving l.s. planes.
Refinement. Refinement of *F*^2^ against ALL reflections. The weighted *R*-factor *wR* and goodness of fit *S* are based on *F*^2^, conventional *R*-factors *R* are based on *F*, with *F* set to zero for negative *F*^2^. The threshold expression of *F*^2^ > σ(*F*^2^) is used only for calculating *R*-factors(gt) *etc*. and is not relevant to the choice of reflections for refinement. *R*-factors based on *F*^2^ are statistically about twice as large as those based on *F*, and *R*- factors based on ALL data will be even larger.

### Fractional atomic coordinates and isotropic or equivalent isotropic displacement parameters (Å^2^)

**Table:**

	*x*	*y*	*z*	*U*_iso_*/*U*_eq_	
O1	−0.1621 (4)	0.29596 (12)	0.33611 (13)	0.0887 (5)	
O2	0.1562 (3)	0.37418 (11)	0.26792 (12)	0.0791 (5)	
O3	−0.2465 (3)	0.53369 (11)	0.15962 (9)	0.0622 (4)	
N1	−0.0008 (3)	0.57463 (11)	0.33181 (9)	0.0424 (4)	
N2	−0.2805 (3)	0.59824 (13)	0.43940 (10)	0.0574 (4)	
N3	−0.0337 (3)	0.37691 (12)	0.31638 (12)	0.0589 (4)	
C1	−0.1098 (3)	0.47575 (13)	0.35252 (12)	0.0464 (4)	
C2	−0.2784 (4)	0.49243 (16)	0.41793 (12)	0.0554 (5)	
H2B	−0.3781	0.4389	0.4442	0.067*	
C3	−0.1137 (4)	0.64611 (14)	0.38648 (11)	0.0485 (4)	
C4	−0.0467 (5)	0.76284 (16)	0.38973 (15)	0.0728 (6)	
H4A	−0.1214	0.7951	0.4404	0.109*	
H4B	−0.1305	0.7965	0.3346	0.109*	
H4C	0.1552	0.7725	0.3963	0.109*	
C5	0.1509 (3)	0.60187 (13)	0.25566 (11)	0.0444 (4)	
H5A	0.3240	0.5598	0.2597	0.053*	
H5B	0.2034	0.6775	0.2595	0.053*	
C6	−0.0235 (3)	0.58087 (13)	0.16507 (12)	0.0451 (4)	
C7	0.1011 (4)	0.62131 (17)	0.08469 (13)	0.0653 (5)	
H7A	−0.0270	0.6069	0.0307	0.098*	
H7B	0.2783	0.5855	0.0810	0.098*	
H7C	0.1326	0.6976	0.0905	0.098*	

### Atomic displacement parameters (Å^2^)

**Table:**

	*U*^11^	*U*^22^	*U*^33^	*U*^12^	*U*^13^	*U*^23^
O1	0.1009 (12)	0.0431 (8)	0.1246 (15)	−0.0117 (8)	0.0248 (10)	0.0121 (8)
O2	0.0790 (10)	0.0538 (9)	0.1115 (13)	0.0122 (7)	0.0387 (9)	−0.0036 (8)
O3	0.0539 (8)	0.0678 (9)	0.0641 (9)	−0.0109 (6)	0.0049 (6)	−0.0016 (6)
N1	0.0423 (7)	0.0430 (8)	0.0437 (8)	−0.0021 (6)	0.0123 (6)	0.0018 (6)
N2	0.0622 (9)	0.0651 (10)	0.0487 (9)	−0.0007 (7)	0.0216 (7)	0.0031 (7)
N3	0.0590 (9)	0.0430 (9)	0.0751 (11)	0.0029 (7)	0.0098 (8)	0.0065 (7)
C1	0.0465 (9)	0.0426 (9)	0.0509 (10)	−0.0010 (7)	0.0098 (7)	0.0075 (7)
C2	0.0541 (10)	0.0610 (12)	0.0530 (11)	−0.0045 (9)	0.0138 (8)	0.0145 (9)
C3	0.0529 (9)	0.0508 (10)	0.0432 (9)	−0.0007 (8)	0.0112 (8)	−0.0011 (7)
C4	0.0993 (16)	0.0555 (12)	0.0684 (14)	−0.0068 (11)	0.0296 (12)	−0.0125 (10)
C5	0.0436 (8)	0.0444 (9)	0.0479 (9)	−0.0045 (7)	0.0159 (7)	0.0003 (7)
C6	0.0457 (9)	0.0395 (9)	0.0518 (10)	0.0047 (7)	0.0124 (7)	−0.0012 (7)
C7	0.0693 (12)	0.0784 (14)	0.0502 (11)	−0.0027 (10)	0.0151 (9)	0.0038 (10)

### Geometric parameters (Å, º)

**Table:**

O1—N3	1.230 (2)	C3—C4	1.481 (3)
O2—N3	1.225 (2)	C4—H4A	0.9600
O3—C6	1.205 (2)	C4—H4B	0.9600
N1—C3	1.358 (2)	C4—H4C	0.9600
N1—C1	1.381 (2)	C5—C6	1.510 (2)
N1—C5	1.457 (2)	C5—H5A	0.9700
N2—C3	1.326 (2)	C5—H5B	0.9700
N2—C2	1.350 (3)	C6—C7	1.486 (3)
N3—C1	1.404 (2)	C7—H7A	0.9600
C1—C2	1.352 (2)	C7—H7B	0.9600
C2—H2B	0.9300	C7—H7C	0.9600
			
C3—N1—C1	104.93 (14)	C3—C4—H4C	109.5
C3—N1—C5	125.87 (14)	H4A—C4—H4C	109.5
C1—N1—C5	128.02 (14)	H4B—C4—H4C	109.5
C3—N2—C2	105.74 (15)	N1—C5—C6	112.47 (13)
O2—N3—O1	122.92 (17)	N1—C5—H5A	109.1
O2—N3—C1	119.63 (15)	C6—C5—H5A	109.1
O1—N3—C1	117.45 (17)	N1—C5—H5B	109.1
C2—C1—N1	107.35 (15)	C6—C5—H5B	109.1
C2—C1—N3	127.87 (16)	H5A—C5—H5B	107.8
N1—C1—N3	124.56 (15)	O3—C6—C7	123.21 (16)
N2—C2—C1	109.97 (15)	O3—C6—C5	121.44 (15)
N2—C2—H2B	125.0	C7—C6—C5	115.35 (14)
C1—C2—H2B	125.0	C6—C7—H7A	109.5
N2—C3—N1	112.01 (16)	C6—C7—H7B	109.5
N2—C3—C4	124.07 (16)	H7A—C7—H7B	109.5
N1—C3—C4	123.86 (16)	C6—C7—H7C	109.5
C3—C4—H4A	109.5	H7A—C7—H7C	109.5
C3—C4—H4B	109.5	H7B—C7—H7C	109.5
H4A—C4—H4B	109.5		
			
C3—N1—C1—C2	−0.39 (18)	C2—N2—C3—N1	−0.6 (2)
C5—N1—C1—C2	−168.41 (15)	C2—N2—C3—C4	−177.73 (19)
C3—N1—C1—N3	−175.31 (16)	C1—N1—C3—N2	0.60 (18)
C5—N1—C1—N3	16.7 (3)	C5—N1—C3—N2	168.96 (14)
O2—N3—C1—C2	−168.38 (18)	C1—N1—C3—C4	177.77 (18)
O1—N3—C1—C2	11.0 (3)	C5—N1—C3—C4	−13.9 (3)
O2—N3—C1—N1	5.5 (3)	C3—N1—C5—C6	−106.10 (18)
O1—N3—C1—N1	−175.14 (16)	C1—N1—C5—C6	59.6 (2)
C3—N2—C2—C1	0.3 (2)	N1—C5—C6—O3	−9.0 (2)
N1—C1—C2—N2	0.1 (2)	N1—C5—C6—C7	171.59 (15)
N3—C1—C2—N2	174.76 (17)		

### Hydrogen-bond geometry (Å, º)

**Table:**

*D*—H···*A*	*D*—H	H···*A*	*D*···*A*	*D*—H···*A*
C2—H2*B*···N2^i^	0.93	2.56	3.361 (2)	144
C5—H5*B*···O2^ii^	0.97	2.57	3.527 (2)	167
C7—H7*B*···O3^iii^	0.96	2.49	3.340 (2)	147

Symmetry codes: (i) −*x*−1, −*y*+1, −*z*+1; (ii) −*x*+1/2, *y*+1/2, −*z*+1/2; (iii) *x*+1, *y*, *z*.
